# Examining bi-directional change in sleep and depression symptoms in individuals receiving routine psychological treatment

**DOI:** 10.1016/j.jpsychires.2023.05.007

**Published:** 2023-07

**Authors:** R. Saunders, Y. Liu, H. Delamain, C. O'Driscoll, S.A. Naqvi, S. Singh, J. Stott, J. Wheatley, S. Pilling, J. Cape, J.E.J. Buckman

**Affiliations:** a CORE Data Lab, Centre for Outcomes Research and Effectiveness (CORE), Research Department of Clinical, Educational, and Health Psychology, UCL, London, United Kingdom; b Barking & Dagenham and Havering IAPT Services – North East London NHS Foundation Trust, London, United Kingdom; c Waltham Forest Talking Therapies - North East London NHS Foundation Trust, London, United Kingdom; d ADAPTlab, Research Department of Clinical Educational and Health Psychology, UCL, London, United Kingdom; e Talk Changes: City & Hackney IAPT Service - Homerton University Hospital NHS Foundation Trust, London, United Kingdom; f Camden and Islington NHS Foundation Trust, London, United Kingdom; g iCope -Camden and Islington Psychological Therapies Services - Camden & Islington NHS Foundation Trust, London, United Kingdom

**Keywords:** Depressive disorder, Sleep disturbance, Psychological treatments, Cross-lagged panel models, Community mental health services

## Abstract

**Background:**

Sleep disturbance is a common symptom of depression. There is conflicting evidence whether improvements in sleep might impact depressive symptoms, or whether treating the core depressive symptoms might improve sleep disturbance. This study explored the bi-directional impact of sleep and depressive symptom change among individuals receiving psychological treatment.

**Methods:**

Session-by-session change in sleep disturbance and depressive symptom severity scores were explored in patients receiving psychological therapy for depression from Improving Access to Psychological Therapies services in England. Bi-directional change in sleep disturbance and depressive symptoms was modelled using random-intercept cross-lagged panel models with items from the PHQ-9.

**Results:**

The sample included 17,732 adults that had received three or more treatment sessions. Both depressive symptoms and sleep disturbance scores decreased. Between initial timepoints, higher sleep disturbance was associated with lower depression scores, but after this point positive cross-lagged effects were observed for both the impact of sleep disturbance on later depressive symptoms, and depressive symptoms on later sleep disturbance scores. The magnitude of effects suggested depressive symptoms may have more impact on sleep than the reverse, and this effect was larger in sensitivity analyses.

**Conclusions:**

Findings provide evidence that psychological therapy for depression results in improvements in core depressive symptoms and sleep disturbance. There was some evidence that depressive symptoms may have more impact on sleep disturbance scores at the next therapy session, than sleep disturbance does on later depressive symptoms. Targeting the core symptoms of depression initially may optimise outcomes, but further research is needed to elucidate these relationships.

## Highlights

• Sessional data on 17,732 patients modelled using random intercept-CLPMs.
• Depression symptom severity and sleep disturbance decreased during treatment.
• Crossed-lagged effects observed for both sleep and core depression symptoms.
• Evidence depression symptoms may have more impact on sleep than vice versa.

## 1 Introduction

Sleep disturbances feature in all diagnostic manuals and guidelines as a symptom of depression and are common across a number of mental disorders (O'Driscoll et al., 2022). Around 90% of individuals with depression are affected by sleep disturbances of one sort or another, with mid-nocturnal insomnia particularly common (Tsuno et al., 2005; Wichniak et al., 2017). For example, it has been observed that around two thirds of people experiencing a severe depressive episode have sleep onset problems, issues with sleep maintenance (frequent awakening) or early morning awakening (delayed or terminal insomnia) (Franzen and Buysse, 2008). Evidence suggests sleep disturbance, for example in response to an adverse event or stressor, is likely to proceed the emergence of mental health issues, potentially by eliciting emotion dysregulation, hyperarousal or negative affect (Freeman et al., 2020). Specifically, insomnia has been identified as a risk factor for both the onset and recurrence of depressive episodes (Buckman et al., 2018; Franzen and Buysse, 2008). Studies exploring the impact of psychological treatments for sleep including studies of individuals with insomnia have observed reductions in depressive symptoms, (Freeman et al., 2020; Troxel et al., 2012), and evidence suggests treating insomnia may reduce the risk of depression occurrence for at least the following year (Cheng et al., 2019). While the above may suggest sleep disturbance is a contributing factor in depression, for many clinicians sleep disturbance is seen as secondary to the mental health issues it occurs alongside (Scott et al., 2017).

Determining whether treating sleep disturbances leads to improvements in the overall severity of depressive symptoms, or whether addressing the core symptoms of depression (anhedonia and low mood) lead to greater improvements (including improving sleep related issues) might inform the overall management of depression and treatment planning including the ordering and selection of interventions. Further, while addressing core depressive symptoms may lead to improvements in sleep as a secondary benefit (Fang et al., 2019), if they do not, then this would be important to note given evidence that sleep disturbances are associated with relapse and recurrence of depression (Buckman et al., 2018) and that treating sleep disturbances might reduce recurrence (Buysse et al., 1996; Henry et al., 2021). Analytical methods to explore the bi-directional relationship between sleep and other depression symptoms during psychological therapy, could improve our understanding of the above factors, and might more easily translate to clinical settings. Sleep problems have been found to be associated with increased anxiety at later time-points among adolescents (Narmandakh et al., 2020), but the session-by-session bi-directional change in depression and sleep disturbance has not been explored in routine psychological therapy.

The aim of the current study was to explore the bi-directional relationship between sleep disturbance and depression symptoms in adults receiving psychological treatment for depression. Specifically, analysis investigated whether changes in sleep precede changes in other depression symptoms, or vice versa, or whether there is a bi-directional relationship.

## 2 Method

### 2.1 Participants and services

The study sample consisted of adult patients from eight Improving Access to Psychological Therapies (IAPT) services in London, which make up part of the North and Central East London IAPT Service Improvement and Research Network (NCEL IAPT SIRN) (Buckman et al., 2021; Saunders et al., 2020). IAPT services provide evidence-based psychological treatments for depression and anxiety disorders across England (Clark, 2018). This national programme was initiated in 2008, with over 1 million patients entering treatment each year (NHS Digital, 2021). IAPT services operate a stepped care model, with patients receiving low intensity (LI) interventions such as guided self-help or group-based interventions based on cognitive behavioural therapy (CBT), and high intensity (HI) interventions including CBT, behavioural activation (BA), or interpersonal therapy (IPT). However, information about specific treatment protocols that were delivered at each session are not available in the data. The decision of which level of intensity (step) and which type of treatment is made jointly between clinicians and patients, with the option to either step-up (or step-down) between intensities depending on need and the degree of symptom change during treatment at the initial step.

Participants for this study were those in the NCEL dataset who had a completed episode of treatment for depression, as indicated by their episode ‘problem descriptor’ (the clinical condition, based on ICD-10 codes, which was the focus on IAPT treatment) between July 2008 and August 2020. In addition, only those meeting ‘caseness’ for depression, defined by the services as scoring 10 or higher on the Patient Health Questionnaire nine-item (PHQ-9; Kroenke et al., 2001) at initial assessment, were included. Further, we only included those who received high intensity interventions, as individuals receiving HI receive more sessions on average, allowing more available data with which to model changes in symptoms. We included patients who had one session of LI only, providing all others were HI, as these LI sessions were typically assessments before allocation to HI treatment. To conduct the specified analyses, we only included individuals for who item level data on the PHQ-9 were available.

### 2.2 Measures

The main measure used for the current analyses was the nine-item Patient Health Questionnaire (PHQ-9; Kroenke et al., 2001). The first two items from the PHQ-9 capture symptoms of ‘anhedonia’ and ‘low mood’, the core symptoms of depression which together make a separate, brief validated screening tool for depression, the PHQ-2 (Arroll et al., 2010). The third item of the PHQ-9 is “trouble falling or staying asleep, or sleeping too much”, and was used to measure sleep disturbances in the current analysis. The PHQ-2 and the sleep item were used in the primary analyses, as the PHQ-2 is a validated measure, but a variable was also created using the eight PHQ-9 items except sleep, which was called the ‘PHQ-8’ and used in sensitivity analyses (described below). The national IAPT programme mandates routine outcome measurement in services meaning that the PHQ-9 was collected at every session, making it an appropriate dataset to measure session-by-session change.

At the initial assessment with services, patients provide data on their age, gender, ethnicity, employment status, and prescription or use of psychotropic medications. Local area deprivation can be calculated from patients’ postal codes using the Index of Multiple Deprivation (IMD), that ranks the relative deprivation of each local area (Department for Communities and Local Government, 2015). For the current analysis, deprivation rank was transformed into quintiles where 1 = most deprived and 5 = least deprived. These were all considered potential confounders in the sensitivity analyses described below.

### 2.3 Statistical analyses

The bi-directional relationship between changes in sleep disturbances and depressive symptoms during psychological treatment was explored using cross-lagged panel models (Kearney, 2017). As the traditional cross-lagged model does not account for construct stability, continual between-person differences might lead to inaccurate results in determining the relationship between the two variables. We therefore used a random-intercept cross-lagged panel model (RI-CLPM) (Hamaker et al., 2015). The inclusion of the random intercept accounts for trait-like stability (within individuals), as a time-invariant factor. This allows individuals to vary around their specific, and relatively constant, trait-level expression of the constructs under examination (Mulder and Hamaker, 2021), focusing on shared group means across time. In addition, following the recommendations of Falkenström et al. (2017) we also conducted a latent curve model with structure residuals (LCM-SR) (Curran et al., 2014) to explore the impact of detrended variables on the primary analyses, and to assess the robustness of effects (see details of the sensitivity analyses below).

For the primary analysis, the PHQ-2 and the sleep item were used as the ‘sleep’ and ‘depressive symptom’ factors. Recent findings indicate that the second, rather than first, session might be better to use as the baseline when modelling change in symptoms during and pre-post treatment in settings such as those from which data were gathered for this study (O'Driscoll et al., 2023). This is because the first appointment is an assessment, whereas the second session is typically the point at which formal treatment starts. We therefore included data from five timepoints, the second appointment with the services up to the sixth, but also demonstrate the impact of using the first session in supplementary analyses (detailed below). Previous analyses have indicated that on average, most change in symptoms during treatment in these settings occurs within the first six sessions (Saunders et al., 2019). The observed mean sleep and depression scores were regressed onto their own individual latent factor (with loadings constrained to one), and these 10 (five sleep and five depression) latent factors were then used to estimate autoregressive and cross-lagged paths. The observed variables' residual variances were set to zero to enable the model structure to account for both within-person and between-person variation (Mulder and Hamaker, 2021). Random intercepts (one for depression and one for sleep) were then added to the model to describe the trait-like differences between patient's variation in these constructs (Narmandakh et al., 2020). The random intercept's covariance also seeks to account for the between-person associations in the two series. The autoregressive paths from one timepoint to the next, within depression or sleep, represent how scores at the previous timepoint predict the following timepoint, for example how severity at the current session predicts severity at the next session. The cross-lagged paths between depression and sleep represent the bidirectional relationships between the constructs and to what extent previous sleep scores predicted following depression scores and how previous depression predicted following sleep scores. The correlation between the residuals of the latent factors depression and sleep at each timepoint represented whether within-person fluctuations in sleep were linked to within-person fluctuations in depression.

**Fig. 1:**
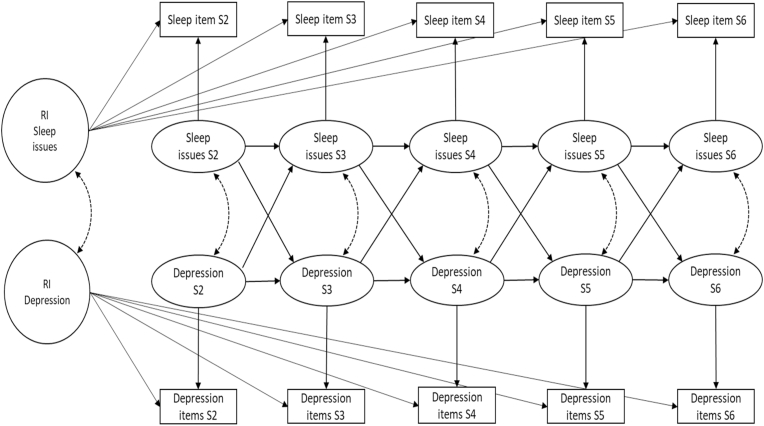
Proposed RI-CLPM model.

The proposed model structure is presented in Fig. 1. Five additional sensitivity analyses were conducted to explore the robustness of the primary findings. The first added gender, age, ethnicity, employment status, local area deprivation, and psychotropic medication usage as covariates in the proposed model. The second sensitivity analysis replaced the PHQ-2 with a measure termed the ‘PHQ-8’, which in the current analysis was the sum of all PHQ-9 items except the sleep item (i.e., scores could range from 0 to 24). This PHQ-8 scale has not been validated, hence is only used as a secondary outcome, but was considered of interest given sleep may impact more than just the core symptoms of depression. The third sensitivity used only the ‘low mood’ question (item 2) of the PHQ-9, to provide a comparison where both the sleep and depression item were scored on a 0–3 scale, therefore with similar likely variance, and how this might impact bi-directional paths. This was referred to as the ‘PHQ-1’ in analyses. The fourth sensitivity analysis replicated the primary model but also included the very first timepoint (T1) in analyses to assess the impact of ignoring the first available measurement and the fifth sensitivity analysis used an alternative approach, the LCM-SR to assess the impact of detrending (Curran et al., 2014).

The model fit of the specified RI-CPLMs were assessed using the root mean square error of approximation (RMSEA), the comparative fit index (CFI), the Tucker-Lewis Index (TLI) and standardised root mean squared residual (SRMR) (Berlin et al., 2014; Geiser, 2013; Schermelleh-Engel et al., 2003). In line with existing guidance, RMSEA and SRMR values below 0.05 were taken to indicate excellent model fit (Jöreskog and Sörbom, 1993) (Byrne, 1998), and values over 0.90 were taken to indicate good fit, with values over 0.95 considered excellent fit for the CFI and TFI values (Hu and Bentler, 1999). Data cleaning and descriptive statistics were conducted in StataCorp (2019) and RI-CLPM models were estimated in Mplus Version 8.3 (Muthén and Muthén, 2020).

## 3 Results

**Fig. 2:**
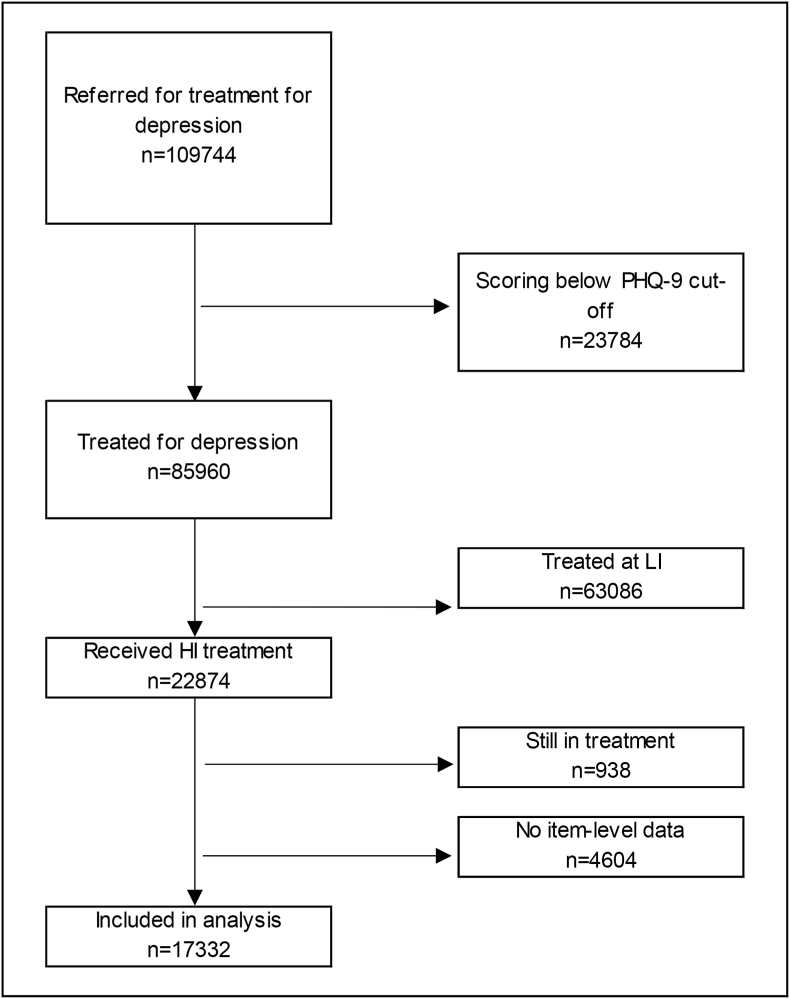
Participant flow diagram.

Fig. 2 shows the participant flow diagram. From n = 519,023 referrals, the majority (79%) were excluded as they did not have depression recorded as their problem descriptor. A total of n = 17,332 individuals were included in the analytic sample.

### 3.1 Descriptive statistics

Descriptive statistics of the sample are presented in Table 1. Most of the sample were female, from white ethnicity groups, not taking psychotropic medication, in employment and residing in a more deprived local area. The most common age category was 25–34 years, with 35–44 years old the next most common and over 65 years the smallest age category (except for missing). Mean scores on the sleep item and the PHQ-2 at each time point are also presented in Table 1. Both sets of scores appear to decrease over all timepoints.

**Table 1:** Descriptive statistics.

Variable	Category	N	%
Age	18–24	2377	13.71
	25–34	5362	30.94
	35–44	3937	22.72
	45–54	3197	18.45
	55–64	1764	10.18
	65+	602	3.47
	Missing	93	0.54
Gender	Male	5313	30.65
	Female	11961	69.01
	Missing	58	0.33
Ethnicity	White	10230	59.02
	Asian	2457	14.18
	Black	2109	12.17
	Mixed	1152	6.65
	Other	805	4.64
	Missing	579	3.34
Psychotropic Medication	Not Taking	9137	52.72
	Taking	6877	39.68
	Missing	1318	7.6
Employment	Employed	11855	68.4
	Unemployed	5230	30.18
	Missing	247	1.43
IMD^a^ quintile	1	5664	32.68
	2	5798	33.45
	3	3201	18.47
	4	1871	10.8
	5	544	3.14
	Missing	254	1.47
Variable	Timepoint	Mean	SD
Sleep	Time 1^a^	2.38	0.85
	Time 2	2.21	0.94
	Time 3	2.07	0.98
	Time 4	1.95	1.00
	Time 5	1.87	1.01
	Time 6	1.81	1.01
PHQ-2	Time 1^a^	4.49	1.40
	Time 2	3.98	1.61
	Time 3	3.68	1.68
	Time 4	3.45	1.72
	Time 5	3.26	1.73
	Time 6	3.14	1.73

*Note*.

Index of Multiple Deprivation.

data from assessment sessions (Time 1) not used in modelling (except specific sensitivity analysis).

### 3.2 Cross-lagged panel modelling

Excellent fit was observed for the primary proposed model including sleep and the PHQ-2 (RMSEA = 0.038, CFI = 0.992, TFI = 0.981, SRMR = 0.027). Correlations between PHQ-2 and sleep item scores at each time point appeared to increase over time (T2 r = 0.187; T3 r = 0.273; T4 r = 0.343; T5 r = 0.394; T6 r = 0.388; all p < 0.001), indicating within-person change in sleep problems was associated with within-person changes in depression scores. Furthermore, the correlation between the random intercepts was r = 0.667 (p < 0.001) indicating a high degree of covariance at the between-person level. This would suggest that individuals with higher scores on the sleep item over the five time periods also reported higher depression symptom severity over the same period.

The autoregressive paths are presented in Table 2 and it was observed that all paths were statistically significant, and increased in magnitude over timepoints. Within-person deviation from the mean value was predictive of scores at the next timepoint. Significant cross-lagged paths were observed at all timepoints between depression and sleep scores, except for the path between PHQ-2 scores at T2 and Sleep at T3 (β = −0.013, p = 0.312) (see Table 2 for details). A negative coefficient was observed between sleep scores at T2 and PHQ-2 scores at T3, indicating that higher sleep scores compared to the mean were associated with lower depression severity (compared to mean depression scores). However, this direction changed from T3 whereby all coefficients were positive. The standardised coefficients were slightly larger for the depression to sleep paths than the sleep to depression paths from T3, which might indicate that changes in depression could be driving changes in sleep score more than the reverse.

**Table 2:** Autoregressive and cross-lagged paths for the primary analysis.

	Predictor	Outcome	Standardised coefficient (β)	p-value
Autoregressive	Sleep T2	Sleep T3	0.138	<0.001
	PHQ2 T2	PHQ2 T3	0.160	<0.001
	Sleep T3	Sleep T4	0.180	<0.001
	PHQ2 T3	PHQ2 T4	0.217	<0.001
	Sleep T4	Sleep T5	0.219	<0.001
	PHQ2 T4	PHQ2 T5	0.295	<0.001
	Sleep T5	Sleep T6	0.256	<0.001
	PHQ2 T5	PHQ2 T6	0.337	<0.001
Cross-lagged	Sleep T2	PHQ2 T3	−0.039	0.003
	PHQ2 T2	Sleep T3	−0.013	0.312
	Sleep T3	PHQ2 T4	0.038	0.002
	PHQ2 T3	Sleep T4	0.058	<0.001
	Sleep T4	PHQ2 T5	0.098	<0.001
	PHQ2 T4	Sleep T5	0.119	<0.001
	Sleep T5	PHQ2 T6	0.115	<0.001
	PHQ2 T5	Sleep T6	0.167	<0.001

### 3.3 Sensitivity analyses

In the first sensitivity analysis, covariates (age group, gender, ethnicity, use of psychotropic medication, employment status and IMD quintile) were added to the model using PHQ-2 scores. Excellent fit was observed for the model on all statistics except for the TFI, where good fit was noted (RMSEA = 0.036, CFI = 0.994, TFI = 0.917, SRMR = 0.012). Autoregressive and cross-lagged paths, presented in Table 3, were very similar to those presented in the main analysis, with statistical significance observed for the same paths. In the second sensitivity analysis, the PHQ-2 was replaced with the PHQ-8, the total of all PHQ-9 items except the sleep item. Excellent fit was observed for the model (RMSEA = 0.043, CFI = 0.992, TFI = 0.982, SRMR = 0.026). The autoregressive and cross-lagged paths are presented in Table 3, and whilst the statistical significance of the paths was the same as with the main analysis, the standardised coefficients were much larger for the depression to sleep path, which might indicate that changes in depression have a bigger impact on sleep scores than the other way around. For the third sensitivity analysis, using just the ‘low mood’ item of the PHQ-9 as a measure of depressive symptoms on a 0–3 scale, and defined as the ‘PHQ-1’ here, excellent fit was also observed (RMSEA = 0.035, CFI = 0.992, TFI = 0.981, SRMR = 0.026). The direction of the coefficients was the same, and the magnitude very similar to that presented in the primary analysis, perhaps indicating that the larger coefficients from depression to sleep were not an artefact of using more items on the measure of depressive symptoms.

**Table 3:** Autoregressive and cross-lagged paths for the sensitivity analyses.

	PHQ-2 with covariates				PHQ-8 (all items except sleep)				PHQ1 (item 2 only)			
	Predictor	Outcome	β	p-value	Predictor	Outcome	β	p-value	Predictor	Outcome	β	p-value
Autoregressive	Sleep T2	Sleep T3	0.144	<0.001	Sleep T2	Sleep T3	0.137	<0.001	Sleep T2	Sleep T3	0.141	<0.001
	PHQ2 T2	PHQ2 T3	0.162	<0.001	PHQ8 T2	PHQ8 T3	0.156	<0.001	PHQ1 T2	PHQ1 T3	0.146	<0.001
	Sleep T3	Sleep T4	0.181	<0.001	Sleep T3	Sleep T4	0.162	<0.001	Sleep T3	Sleep T4	0.182	<0.001
	PHQ2 T3	PHQ2 T4	0.215	<0.001	PHQ8 T3	PHQ8 T4	0.326	<0.001	PHQ1 T3	PHQ1 T4	0.171	<0.001
	Sleep T4	Sleep T5	0.215	<0.001	Sleep T4	Sleep T5	0.175	<0.001	Sleep T4	Sleep T5	0.228	<0.001
	PHQ2 T4	PHQ2 T5	0.293	<0.001	PHQ8 T4	PHQ8 T5	0.440	<0.001	PHQ1 T4	PHQ1 T5	0.231	<0.001
	Sleep T5	Sleep T6	0.251	<0.001	Sleep T5	Sleep T6	0.201	<0.001	Sleep T5	Sleep T6	0.226	<0.001
	PHQ2 T5	PHQ2 T6	0.337	<0.001	PHQ8 T5	PHQ8 T6	0.501	<0.001	PHQ1 T5	PHQ1 T6	0.267	<0.001
Cross-lagged	Sleep T2	PHQ2 T3	−0.034	0.007	Sleep T2	PHQ8 T3	−0.043	0.002	Sleep T2	PHQ1 T3	−0.029	0.023
	PHQ2 T2	Sleep T3	−0.006	0.625	PHQ8 T2	Sleep T3	−0.004	0.819	PHQ1 T2	Sleep T3	−0.020	0.109
	Sleep T3	PHQ2 T4	0.040	0.001	Sleep T3	PHQ8 T4	0.041	0.001	Sleep T3	PHQ1 T4	0.030	0.001
	PHQ2 T3	Sleep T4	0.056	<0.001	PHQ8 T3	Sleep T4	0.122	<0.001	PHQ1 T3	Sleep T4	0.046	<0.001
	Sleep T4	PHQ2 T5	0.095	<0.001	Sleep T4	PHQ8 T5	0.065	<0.001	Sleep T4	PHQ1 T5	0.103	<0.001
	PHQ2 T4	Sleep T5	0.114	<0.001	PHQ8 T4	Sleep T5	0.204	<0.001	PHQ1 T4	Sleep T5	0.096	<0.001
	Sleep T5	PHQ2 T6	0.108	<0.001	Sleep T5	PHQ8 T6	0.070	<0.001	Sleep T5	PHQ1 T6	0.107	<0.001
	PHQ2 T5	Sleep T6	0.164	<0.001	PHQ8 T5	Sleep T6	0.253	<0.001	PHQ1 T5	Sleep T6	0.146	<0.001

*Note*. β = Standardised coefficient.

The fourth sensitivity analysis included the first timepoint, when the first assessment took place (T1) and the results are presented in Supplementary Table S1. The model included T1 to T6, for the sample and showed poorer model fit than the primary analysis (RMSEA = 0.053; CFI = 0.975; TFI = 0.954; SRMR = 0.052). It was noted that the initial autoregressive path between PHQ-2 at T1 and T2 was not significant, but otherwise the findings mirrored those of the main analyses (presented in Table 2), especially the magnitude of cross-lagged paths observed. In the final sensitivity analysis an LCM-SR was constructed using the PHQ-2 and the sleep disturbance item, with the associations presented in Supplementary Table S2. The model fit was improved compared to the original model (RMSEA = 0.023; CFI = 0.995; TFI = 0.993; SRMR = 0.028), and coefficients for both the autoregressive and cross-lagged associations were reduced when compared to the primary model. This is anticipated given the LCM-SR's consideration of potential unmeasured confounders, but it was noted that cross-lagged associations were still present in the model, and with the depression to sleep coefficients larger than those for sleep to depression, as observed in the primary model. One difference was that the initial path (T2- > T3) between sleep and depression was not statistically significant in LCM-SR, compared to the negative coefficient observed in the primary model, and that the initial path between depression and sleep was statistically significant.

## 4 Discussion

This study explored the bi-directional effect of change in sleep disturbance and change in depressive symptoms during the initial sessions of psychological treatment for depression. Findings suggested strong autoregressive pathways within sleep and depressive symptom change, as well as a bi-directional relationship. Sensitivity analysis in which models: 1) controlled for potential confounders, 2) used further items on the PHQ-9 in addition to the core symptoms, and 3) used only the ‘low mood’ item, identified the same pathways and supported the findings of the primary analysis. Standardised coefficients were larger for the association between depression and later sleep scores, than for sleep to later depression, especially for the sensitivity analysis using more items from the PHQ-9.

That a bi-directional relationship between sleep disturbance and depressive symptoms scores was observed from session three supports suggestions that there is not a simple cause and effect relationship between them (Fang et al., 2019). The size of the standardised coefficients, particularly for the sensitivity analysis using all eight remaining items of the PHQ-9 (after removing the sleep item), may support the notion that change in depression scores are more strongly associated with subsequent change in sleep disturbance than the other way around. It might be the case that routinely delivered psychological therapies for depression, such as those provided by these services, are more targeted at core symptoms of depression over sleep disturbance, or potentially that therapists are more likely to treat sleep as a non-specific symptom and therefore focus on other symptoms, at least earlier in the process of therapy (Freeman et al., 2020). However, all of the patients in this study were treated for depression such that all had at least one of the core depressive symptoms at baseline, but not all patients reported sleep disturbance, and it appears as though changes in the core depressive symptoms led to later fluctuations in sleep disturbance whether or not it was directly targeted in treatment.

Alternatively, the increased magnitude of the association in the sensitivity analysis using the eight items of the PHQ-9 might point to a stronger association between improvement in non-core symptoms of depression and subsequent change in sleep symptoms than the association between changes in the core symptoms and later change in sleep symptoms. Indeed, other studies have highlighted strong paths suggesting non-core depressive symptoms precede change in anhedonia (O'Driscoll et al., 2021, 2022). Analysis exploring cross-lagged associations in data from groups of individuals treated explicitly for insomnia may elicit different paths. It may also be that sleep symptoms take longer to change than depressive symptoms from routinely delivered psychological treatments, which may explain why the impact of depressive symptoms on sleep disturbance at the next session is of a larger magnitude than the opposite. This would potentially be in-line with evidence from studies that have reported improvements in depressive symptoms at the end of treatment but not in sleep disturbance, and that as such, residual post-treatment sleep disturbance symptoms are associated with an increased risk of recurrence of depression (Buckman et al., 2018; Nutt et al., 2008).

Higher sleep disturbance at session two was associated with lower depression symptoms at session three in all models. Early change in therapy is considered a key indicator of success in treatment for depression, and on average occurs by session three or four (Lambert, 2013), although it can be later for certain groups (Saunders et al., 2019). The findings between session two and three may therefore be due to higher sleep being associated with higher depression at the same timepoint, and therefore a bigger decrease in scores by the next timepoint, as this is where the most decline happens. However, detrending the model through the use of the LCM-SR changed this effect, so that sleep at T2 was not statistically associated with depression at T3. As a result, further evaluation of this finding, especially with more sensitive measures of sleep disturbance might help elucidate this relationship.

### 4.1 Limitations

Within IAPT services, a range of therapies are recommended to treat depression (Clark, 2018). The current study did not differentiate patients by therapy type and so it is not clear how individual therapies are associated with the treatment of sleep and depression. Importantly, the exact nature of the therapeutic approaches used in each session are not recorded in detail, so future work might explore whether certain techniques (e.g. sleep hygiene or behavioural therapy for insomnia) are associated with greater change in sleep disturbance and the subsequent effect of this on depressive symptoms. Whilst we included individuals who were treated for depression, we did not exclude people who have a comorbidity of depression and anxiety (or another mental health issue), and results may be different for individuals without comorbid anxiety, although these individuals would be less reflective of routine treatment services. The study only includes individuals with five PHQ-9 assessments after their first initial assessment, which means patients with PHQ-9 scores beyond this were not analysed and so later changes in their sleep symptoms were not investigated here. Further, the study uses the single PHQ-9 item to capture sleep disturbance where responses are provided on a 0–3 scale, thereby limiting the range of scores. The item covers sleep-onset insomnia, sleep-maintenance insomnia as well as prolonged sleep duration. The analyses here were therefore unable to differentiate the type of sleep disturbance experienced by patients in this study, and it may be the case that different effects would be found if such an investigation were possible Future work may seek to use a more detailed sleep measure such as the 19-item Pittsburgh Sleep Quality Index (PSQI (Buysse et al., 1989); that captures wider array of variables such as sleep disturbance, subjective quality, duration, and latency. The analytic approach did not include unmeasured time-varying factors that could be potential confounders of the observed associations, and findings from the LCM-SR highlight the potential role of such confounders in these associations. Further, although we were able to include a number of important potential confounders in the models there were many others that could not be included as they are not routinely measured in IAPT services. Having experienced negative life events, less stable home environments and financial hardship have all been associated with poorer outcomes from treatment for depression and are likely to impact sleep disturbance (Buckman et al., 2022a; Buckman et al., 2022b). The inclusion of these variables in future analyses might elucidate more about the independence of the presented effects. Lastly, the models presented estimate changes using means over the sample, but there is potential for heterogenous subgroups of individuals whose sleep (and depressive symptom) trajectories do not follow the same declining trajectory, and instead either do not change or show a deterioration. The use of modelling approaches, such as growth mixture modelling (Muthén, 2001; Muthén et al., 2002) might further elucidate the process of change in sleep disturbances during psychological treatment.

### 4.2 Implications

The study has demonstrated a bi-directional relationship between changes in sleep disturbances and depressive symptoms during psychological treatments for depression. Whilst psychological treatments for depression routinely target the core symptoms of depression, these findings highlight the potential role of sleep disturbance in prognosis and suggest that targeting sleep disturbances when they are present, might improve outcomes. These results indicated that the impact of depressive symptoms on sleep may be larger than sleep disturbance on depression, but this difference was small in the primary analyses, and therefore further work to understand these differences is needed especially using a more sensitive measure of sleep. Given that patients with residual depressive symptoms are much more likely to relapse than those that experience a full-remission, and sleep disturbances are among the most common residual symptoms following treatment (Wichniak et al., 2017), monitoring sleep disturbance issues during treatment might optimise both short and long-term outcomes.

### 4.3 Conclusion

Using a large dataset of individuals receiving psychological treatments for depression in a naturalistic setting it was observed that depressive symptoms and sleep disturbances were highly correlated, and scores at individual time points were associated with within-person changes in subsequent time points. Autoregressive effects were particularly strong, and bi-directional cross-lagged effects existed between sleep disturbances and depressive symptoms from session three onwards. It was observed that the effects for depression symptom severity on later sleep scores were bigger than for the reverse relationship, which may suggest that those delivering treatments for depression should target sleep disturbance alongside core depressive symptoms, as this is likely to optimise outcomes. Future research should explore these bi-directional effects within specific modalities of therapy, or between them, to elucidate whether specific approaches are driving these relationships.

## CRediT authorship contribution statement

Rob Saunders: Conceptualization, Methodology, Formal analysis, Investigation, Writing – original draft, Project administration. Yiting Liu: Conceptualization, Methodology, Formal analysis, Investigation, Writing – original draft. Henry Delamain: Methodology, Writing – review & editing. Ciaran O'Driscoll: Methodology, Writing – review & editing. Syed Ali Naqvi: Data curation, Writing – review & editing. Satwant Singh: Data curation, Writing – review & editing. Joshua Stott: Methodology, Writing – review & editing. Jon Wheatley: Data curation, Writing – review & editing. Stephen Pilling: Data curation, Writing – review & editing. John Cape: Conceptualization, Writing – review & editing. Joshua E.J. Buckman: Conceptualization, Methodology, Funding acquisition, Writing – review & editing.

## Financial support

This work was supported by the 10.13039/501100000272National Institute for Health Research (NIHR) 10.13039/501100012621University College London Hospitals 10.13039/501100012621Biomedical Research Centre, 10.13039/501100000765University College London (UCL) and the 10.13039/100010269Wellcome Trust (Grant Code, 201292/Z/16/Z).

## Declaration of competing interest

All authors declare that there are no conflicts of interest.
