# Supplementary material for: Examining bi-directional change in sleep and depression symptoms in individuals receiving routine psychological treatment
Source: J Psychiatr Res. 2023 Jul;163:1–8. doi: 10.1016/j.jpsychires.2023.05.007 (PMC10643991; doi:10.1016/j.jpsychires.2023.05.007)
Supplement: Multimedia component 1 [file mmc1.docx]

Supplementary materials to:

**Examining bi-directional change in sleep and depression symptoms in individuals receiving routine psychological treatment**

**Content:**

**Supplementary Table S1: RI-CLPM results including baseline assessment (Page 2)**

**Supplementary Table S2: LCM-SR results for primary proposed model (Page 3)**

**Supplementary Table S1: RI-CLPM results including baseline assessment**

|  | Predictor | Outcome | Standardised coefficient (β) | p-value |
| --- | --- | --- | --- | --- |
| Autoregressive | Sleep T1 | Sleep T2 | 0.048 | 0.001 |
|  | PHQ2 T1 | PHQ2 T2 | 0.022 | 0.154 |
|  | Sleep T2 | Sleep T3 | 0.233 | <0.001 |
|  | PHQ2 T2 | PHQ2 T3 | 0.329 | <0.001 |
|  | Sleep T3 | Sleep T4 | 0.274 | <0.001 |
|  | PHQ2 T3 | PHQ2 T4 | 0.392 | <0.001 |
|  | Sleep T4 | Sleep T5 | 0.301 | <0.001 |
|  | PHQ2 T4 | PHQ2 T5 | 0.423 | <0.001 |
|  | Sleep T5 | Sleep T6 | 0.314 | <0.001 |
|  | PHQ2 T5 | PHQ2 T6 | 0.437 | <0.001 |
| Cross-lagged | Sleep T1 | PHQ2 T2 | -0.069 | <0.001 |
|  | PHQ2 T1 | Sleep T2 | -0.065 | <0.001 |
|  | Sleep T2 | PHQ2 T3 | 0.079 | <0.001 |
|  | PHQ2 T2 | Sleep T3 | 0.123 | <0.001 |
|  | Sleep T3 | PHQ2 T4 | 0.120 | <0.001 |
|  | PHQ2 T3 | Sleep T4 | 0.191 | <0.001 |
|  | Sleep T4 | PHQ2 T5 | 0.153 | <0.001 |
|  | PHQ2 T4 | Sleep T5 | 0.206 | <0.001 |
|  | Sleep T5 | PHQ2 T6 | 0.154 | <0.001 |
|  | PHQ2 T5 | Sleep T6 | 0.234 | <0.001 |

Model fit statistics: RMSEA = 0.053; CFI = 0.975; TFI = 0.954; SRMR = 0.052.

**Supplementary Table S2: LCM-SR results for primary proposed model**

|  | Predictor | Outcome | Standardised coefficient (β) | p-value |
| --- | --- | --- | --- | --- |
| Autoregressive | Sleep T2 | Sleep T3 | 0.140 | <0.001 |
|  | PHQ2 T2 | PHQ2 T3 | 0.174 | <0.001 |
|  | Sleep T3 | Sleep T4 | 0.147 | <0.001 |
|  | PHQ2 T3 | PHQ2 T4 | 0.176 | <0.001 |
|  | Sleep T4 | Sleep T5 | 0.144 | <0.001 |
|  | PHQ2 T4 | PHQ2 T5 | 0.183 | <0.001 |
|  | Sleep T5 | Sleep T6 | 0.149 | <0.001 |
|  | PHQ2 T5 | PHQ2 T6 | 0.180 | <0.001 |
| Cross-lagged | Sleep T2 | PHQ2 T3 | 0.019 | 0.068 |
|  | PHQ2 T2 | Sleep T3 | 0.025 | 0.022 |
|  | Sleep T3 | PHQ2 T4 | 0.022 | 0.019 |
|  | PHQ2 T3 | Sleep T4 | 0.030 | 0.003 |
|  | Sleep T4 | PHQ2 T5 | 0.027 | 0.003 |
|  | PHQ2 T4 | Sleep T5 | 0.036 | <0.001 |
|  | Sleep T5 | PHQ2 T6 | 0.034 | 0.018 |
|  | PHQ2 T5 | Sleep T6 | 0.046 | 0.002 |

Model fit statistics: RMSEA = 0.023; CFI = 0.995; TFI = 0.993; SRMR = 0.028.
